# Nailfold capillaroscopy in diabetic retinopathy: microvascular insights, clinical value, and translational challenges — a narrative review

**DOI:** 10.3389/fendo.2026.1866310

**Published:** 2026-08-13

**Authors:** Yi-Mai Zhou, Lu-Sha Cen

**Affiliations:** 1 The First Clinical Medical College, Zhejiang Chinese Medical University, Hangzhou, Zhejiang, China; 2 Department of Ophthalmology, The First Affiliated Hospital of Zhejiang Chinese Medical University (Zhejiang Provincial Hospital of Chinese Medicine), Hangzhou, Zhejiang, China

**Keywords:** avascular area, capillary density, diabetic microangiopathy, diabetic retinopathy, microcirculation, nailfold capillaroscopy, translational research

## Abstract

Diabetic retinopathy (DR) is a leading microvascular complication of diabetes mellitus and a major cause of preventable vision loss. DR is now viewed as a manifestation of systemic diabetic microangiopathy rather than an isolated ocular disorder. Retina-centered assessment may not fully capture systemic microvascular burden, particularly where access to specialized retinal imaging is constrained. Nailfold capillaroscopy (NFC) enables direct, non-invasive visualization of peripheral capillary morphology and has been investigated as a marker of systemic microvascular abnormalities in diabetes. This narrative review examines shared mechanisms of retinal and peripheral microvascular injury and summarizes evidence linking NFC abnormalities to DR presence and severity. The available cross-sectional evidence was used to develop an exploratory three-tiered, pattern-based framework. Background features include capillary tortuosity, mild dilation, and microhemorrhages, which have been associated with prevalent DR but show limited consistency in discriminating current severity. Auxiliary features include neoangiogenic patterns, megacapillaries, and crossing capillaries, while the proposed core pattern comprises reduced capillary density and avascular areas. Among these parameters, avascular areas showed the most consistent pooled association with current DR severity. Selected single-cohort analyses reported AUC values above 0.80 for cross-sectional discrimination of prevalent DR or PDR; however, these estimates have not been prospectively or externally validated. In one cross-sectional cohort, a capillary density cutoff of <7.5 capillaries/mm yielded 95% sensitivity but only 65% specificity for prevalent DR, while a multi-parameter composite score showed an AUC of 0.745 in another cohort. The cumulative layering model and three-tiered pattern-based framework synthesize these findings for exploratory analysis of cross-sectional NFC–DR associations and discrimination of DR severity. However, the available evidence is predominantly cross-sectional and heterogeneous, with variability in imaging equipment, morphological definitions, sample sizes, and confounder adjustment. The framework is therefore hypothesis-generating and is not suitable at present for guiding routine clinical management or ophthalmology referral. Its longitudinal relevance requires prospective validation. NFC remains investigational. Further evaluation requires standardized imaging protocols, automated quantitative analysis, and large prospective studies.

## 1 Introduction

Diabetic retinopathy (DR) affects approximately one-third of adults with diabetes worldwide and remains a leading cause of preventable blindness in the working-age population (1, 2). Conceptually, understanding of DR has shifted markedly over the past decade: rather than an isolated ocular complication, DR is increasingly recognized as a local expression of systemic diabetic microangiopathy, characterized by parallel endothelial dysfunction, capillary rarefaction, and impaired perfusion across multiple vascular beds (3). Several studies have reported detectable peripheral microvascular abnormalities in individuals without clinically evident retinal lesions (4–6), suggesting that peripheral assessment may capture an additional dimension of systemic microvascular burden.

Current DR screening and monitoring rely predominantly on ophthalmic imaging modalities, including fundus photography, optical coherence tomography (OCT), and optical coherence tomography angiography (OCTA) (7, 8). These tools are central to retinal assessment but require specialized equipment and trained personnel, which may limit their use in primary-care and resource-constrained settings (9, 10). Nailfold capillaroscopy (NFC) offers a relatively accessible method for direct *in vivo* imaging of peripheral capillary morphology (11). Recent observational studies and meta-analyses have reported associations between nailfold abnormalities and prevalent DR, although prospective validation is lacking (12). The available evidence mainly addresses cross-sectional association with prevalent DR and discrimination of current severity; longitudinal prediction of DR onset or progression remains uncertain. To date, no universally accepted framework links observable NFC phenotypes to the underlying biological mechanisms or cross-sectionally observed patterns of DR. This review summarizes shared mechanisms of retinal and peripheral microvascular injury, evaluates associations between NFC abnormalities and established DR, and proposes the cumulative layering model and pattern-based framework for prospective evaluation.

## 2 Narrative review methodology

Structured searches were conducted in PubMed, Embase, Scopus, and Web of Science from database inception to 27 April 2026 using the following core search string: (“diabetes mellitus” OR “diabetic” OR “T1DM” OR “T2DM”) AND (“diabetic retinopathy” OR “retinopathy” OR “NPDR” OR “PDR” OR “diabetic macular edema”) AND (“nailfold capillaroscopy” OR “nailfold videocapillaroscopy” OR “nailfold video capillaroscopy” OR “nailfold microscopy” OR “nailfold microcirculation” OR “nailfold dermatoscopy” OR “nailfold dermoscopy” OR “USB microscopy”). The search syntax was adapted as appropriate for each database. Eligible evidence comprised human observational studies reporting NFC findings in relation to the presence or current severity of DR, together with relevant systematic reviews or meta-analyses synthesizing such studies. Only full-text publications providing sufficient methodological and outcome data to permit study-level appraisal were eligible. Studies conducted in non-diabetic populations were considered only when they provided relevant technical information and were not treated as direct evidence regarding DR. Animal and *in vitro* studies, duplicate records, conference abstracts without sufficient full-text data, and studies without relevant NFC or DR outcomes were excluded. Two reviewers independently screened titles and abstracts and subsequently assessed potentially eligible full-text articles against the prespecified eligibility criteria. Disagreements were resolved through discussion and consensus. All studies meeting the eligibility criteria were included in the narrative synthesis. The final synthesis comprised 21 eligible publications, including one meta-analysis and 20 observational studies. The study populations, DR grading, principal findings, analytical methods, and methodological considerations of all included studies are summarized in **Supplementary Table 1**. No protocol was registered, and no *de novo* quantitative synthesis was performed; between-study heterogeneity was assessed qualitatively.

## 3 Pathophysiological basis: systemic microvascular injury in diabetes

Chronic hyperglycemia triggers a network of metabolic and cellular disturbances that drive systemic microvascular injury and underlie diabetic complications, including DR (3). Functional abnormalities may precede structural remodeling across several microvascular beds (13).

At the molecular level, hyperglycemia-induced oxidative stress and chronic low-grade inflammation are central to diabetic microvascular dysfunction (14, 15). Several interconnected pathways are implicated: the AGEs–RAGE axis amplifies oxidative stress and impairs endothelial function (16, 17); PKC activation modulates vascular permeability and enhances VEGF-mediated pro-angiogenic signaling (18); and the polyol pathway compromises antioxidant defenses via NADPH depletion (19). These pathways converge to disrupt endothelial integrity, impair vascular autoregulation, and promote capillary leakage and occlusion — changes conserved in both retinal and peripheral vascular beds (20). Concurrently, ROS- and AGE-driven NF-κB activation sustains endothelial inflammation, leukocyte adhesion, and microvascular obstruction, further contributing to barrier disruption, tissue hypoxia, and microvascular rarefaction (21–23).

At the tissue level, early endothelial dysfunction and impaired vasoreactivity progress to increased vascular resistance and reduced perfusion (24). Sustained injury leads to structural remodeling — pericyte loss, basement membrane thickening, capillary dilation, and eventual capillary dropout (25, 26) — culminating in hypoxia-driven pathological angiogenesis in advanced stages (27). Systemic factors, including diabetes duration, glycemic burden, hypertension, and dyslipidemia, further modulate the extent and rate of this process. The concept of “vascular memory” describes persistent microvascular effects of cumulative hyperglycemic exposure despite subsequent improvement in glycemic control (28).

These mechanisms can produce capillary alterations, including basement membrane thickening, pericyte loss, capillary rarefaction, and compensatory angiogenesis, in retinal and peripheral vascular beds. Their visibility in peripheral tissues provides a rationale for studying NFC as a marker of systemic microvascular injury.

## 4 Nailfold capillaroscopy: technical principles and assessment domains

NFC has been used as a non-invasive method for evaluating microvascular alterations associated with diabetes (12). Because nailfold capillaries run parallel to the skin surface, NFC permits direct *in vivo* visualization of the capillary loop without tissue sampling (29).

Available devices range from high-resolution nailfold videocapillaroscopes (NVC, ×200–×600 magnification), used as a reference method (30), to lower-cost alternatives such as handheld dermatoscopes and USB digital microscopes, which improve accessibility in resource-limited settings (31). NFC abnormalities can be systematically assessed across several key domains (32): general capillary characteristics (alignment, density, tortuosity), microvascular morphology (proportion of malformed capillaries, diameter variability, loop irregularity), microcirculatory dynamics (red blood cell flow velocity, perfusion uniformity), and pericapillary features (microhemorrhages, exudative changes, perivascular edema) (33). These domains describe structural and functional aspects of the microcirculation (29). Imaging devices, evaluation methods, and standardization protocols vary substantially across studies (34) — issues addressed later in this review.

**Table 1** compares device configurations, reported magnifications, strengths, limitations, and current evidence in diabetes and DR across four NFC imaging modalities. **Table 2** presents morphological features and their descriptive definitions of the principal abnormal nailfold capillary morphologies evaluated in the studies.

**Table 1:** Comparison of four nailfold capillaroscopy devices for diabetic retinopathy assessment.

Modality	Devices & magnification	Strengths and limitations	Evidence in diabetes & DR
Dedicated Nailfold Videocapillaroscopy (NVC) (35–40)	NVC systems (models NR), ×200 (35, 37); AlphaDoc 3.2, ×100/×200 (36); Videocap, ×200 (38); Optilia Video Capillaroscope, ×200 (39); Picocap, ×300 (40)	Strengths: High-resolution digital imaging; structured qualitative/semiquantitative assessment; blinded image archiving and serial comparison. Limitations: High cost and operator-training requirements; heterogeneous acquisition, magnification, and grading protocols.	Cross-sectional studies associated tortuous, crossed, ectatic, and avascular capillaries with prevalent DR, although findings were inconsistent and individual markers showed limited discrimination (35, 37, 38). Abnormalities also occurred without clinical DR (36), and a CNN discriminated diabetes better than DR (AUROC 0.84 vs 0.60) (39). No longitudinal predictive validity has been established.
Automated Quantitative Capillary Microscopy (41)	Kekkan-Bijin SC-10 with Capillary Analysis System, field of view 500 × 700 μm; magnification NR (41)	Strengths: Automated quantification of capillary count, length, width, and turbidity; rapid continuous measurements within a standardized field. Limitations: Proprietary platform with limited cross-platform validation; non-interchangeable field-of-view metrics; sensitivity to image quality, field selection, and algorithm performance.	Capillary length discriminated prevalent DR/PDR (AUROC 0.83), improving to 0.89 when combined with systemic indicators; prospective validation and clinical cutoffs are lacking (41).
Handheld Smartphone-Coupled Dermatoscopy (42–45)	DermLite DL4, ×10/×50 (42, 44) or ×12–×14/×50 (43); DermLite DL3N, ×10 (45)	Strengths: Portable and accessible; supports wide-field assessment, smartphone image capture, and semiquantitative grading. Limitations: Lower resolution and limited dimensional quantification compared with NVC; reduced image visibility in darker skin phototypes.	Cross-sectional data associate avascular/bushy capillaries and composite NFC scores with DR severity and DME, with poorer imaging in darker skin (42, 43). A composite score yields modest DR discrimination (AUROC = 0.745; 51.85% sensitivity, 95.65% specificity), unsuitable for independent screening (44). Unified protocols and external validation are absent.
Low-Cost USB Digital Capillary Microscopy (31, 46, 47)	XCSOURCE TE389, 2–6 μm/pixel (31); Dino-Lite Edge AM7515MZT, ×20–×220 (46); Dino-Lite/DinoCapture 2.0, ×500, recalibrated to ~×200 (47).	Strengths: Low-cost and portable; supports digital image/video capture, storage, repeated review, and exploratory measurement. Limitations: Heterogeneous optics, calibration, and software; non-comparable nominal magnification; image artifacts limit cross-device reproducibility.	More NFC lesions are observed in DR patients (46); capillary anomalies are numerically elevated in DR without statistical intergroup differences (47). No USB microscope has validated diagnostic thresholds or prospective data for incident or progressive DR.

AUROC, area under the receiver operating characteristic curve; CNN, convolutional neural network; DME, diabetic macular edema; DR, diabetic retinopathy; NFC, nailfold capillaroscopy; NR, not reported; NVC, nailfold videocapillaroscopy; PDR, proliferative diabetic retinopathy. Magnification, field of view, and image resolution are reported as described in the original studies and are not directly interchangeable across platforms because of differences in optics, calibration, and image-processing software. Device categories may overlap because some Dino-Lite systems have been described as dermatoscopes, USB microscopes, or videocapillaroscopy devices. Evidence is predominantly cross-sectional; reported AUROCs, sensitivities, and specificities reflect discrimination of prevalent DR rather than longitudinal prediction. Ref. (31) investigated systemic sclerosis and is included only as technical evidence for USB microscopy.

**Table 2:** Morphological features of nailfold capillaries in capillaroscopy.

Morphological feature	Description
Dilated apical segment	Focal dilatation confined predominantly to the apical portion of the capillary loop, with relatively preserved limb caliber.
Dilated capillary (ectasia)	Diffuse enlargement of the loop, typically involving the arterial limb, venous limb, and apex, with a diameter greater than normal but below the threshold for a giant capillary.
Giant capillary (megacapillary)	Marked, homogeneous capillary enlargement, usually defined as a loop diameter exceeding 50 μm or approximately 10-fold the normal caliber.
Meandering capillary (crossed-over vessel)	Capillary loop showing broad self-crossing or serpentine deviation along its course while maintaining overall loop continuity.
Tortuous capillary	Capillary loop with exaggerated curvature, repeated twisting, or coiling of one or both limbs, without the disorganized branching pattern of neoangiogenesis.
Neoangiogenesis	Bushy capillary (48)**:**A capillary loop showing multiple fine sprouting buds or clustered short branches, giving a bush-like appearance.
	Ramified capillary (branched capillary) (49): A capillary loop showing branching or arborization from the main axis, often with irregular secondary branches, consistent with neoangiogenesis.
	Bizarre capillary (48):A markedly distorted, asymmetrical, or irregularly configured capillary loop that cannot be readily classified into conventional morphologic categories.
Receding capillary	Capillary located abnormally proximally relative to the distal row, suggesting regression or remodeling of the capillary bed.
Avascular area (49, 50)	Absence of two or more consecutive distal-row capillaries, forming a distinct capillary-free zone.

This table defines key nailfold capillary abnormalities observed in microvascular dysfunction-related conditions, such as systemic sclerosis and diabetes mellitus. It describes features including dilated segments, capillary ectasia, giant capillaries, meandering/tortuous/angulated forms, neoangiogenesis patterns (bushy, ramified, bizarre), capillary recession, avascular areas, and microhemorrhages, providing concise definitions for each morphological characteristic.

Clinical studies have reported associations between nailfold and retinal microvascular abnormalities, including observations in some patients without clinically apparent DR, supporting further investigation of NFC as a potential correlate of systemic microvascular impairment (35, 42).

## 5 Clinical evidence: NFC abnormalities and diabetic retinopathy

### 5.1 Overall association: NFC abnormalities and DR

Several observational studies and a recent meta-analysis have reported associations between selected NFC abnormalities and prevalent DR. Pooled analyses suggest that several morphological abnormalities, including microhemorrhages, avascular areas, capillary tortuosity, and neoangiogenesis, are more frequent in patients with prevalent DR than in those without DR, although individual studies have reported heterogeneous or null findings (12, 35).

Beyond structural alterations, functional microvascular impairment parallels these changes. Dynamic capillaroscopy studies have documented reduced capillary blood flow velocity and prolonged time-to-peak perfusion in some patients with diabetes but without clinically apparent DR at the time of assessment. Associations have also been reported between hemodynamic dysfunction, capillary tortuosity, and current DR severity, while cutaneous microvascular leakage has been observed in individuals without retinal angiographic abnormalities (51). These findings suggest that peripheral abnormalities may coexist with diabetes before clinically apparent DR, but their temporal relationship with retinal disease requires longitudinal evaluation.

Complementary videocapillaroscopy studies extend these findings, demonstrating elevated capillary abnormality scores in both Type 1 and Type 2 diabetes, with more severe derangements in Type 1 disease (36). These studies reported associations between NFC abnormalities and current DR severity, although the extent of adjustment for clinical confounders varied. NFC abnormalities were also observed in some patients without clinical retinopathy, supporting their evaluation as potential markers of systemic diabetic microangiopathy (36, 37, 51, 52).

**Supplementary Table 1** summarizes all 21 eligible publications, comprising one meta-analysis and 20 observational studies, together with their population characteristics, principal findings, and methodological considerations.

### 5.2 Morphological parameters: discriminative value for DR

The clinical relevance of NFC abnormalities depends strongly on the endpoint under evaluation. Three outcomes are considered separately: association with established DR in contemporaneous observations, discrimination among current DR severity grades, and longitudinal prediction of incident or progressive DR. Based on consistency and biological plausibility, the findings are organized into core parameters, auxiliary markers, and supplementary features, providing a conceptual structure for interpreting static observational associations and designing prospective analyses.

#### 5.2.1 Core parameters: capillary density and avascular areas

Reduced capillary density and avascular areas are among the NFC features most consistently associated with prevalent DR or current DR severity. Okabe et al. reported that quantitative nailfold capillary measures, including count, length, width, and turbidity, differed according to the presence and current severity of DR (41). Elghany et al. reported lower capillary density and greater capillary diameter in patients with DR than in those without DR; in a model restricted to capillaroscopic variables, capillary density remained associated with prevalent DR (53). A meta-analysis by Yan et al. identified avascular areas as the only NFC parameter significantly associated with DR severity grading, whereas tortuosity and microhemorrhages did not differentiate across severity stages (12). Avascular areas were more frequent in PDR than in NPDR in several cross-sectional studies and showed the most consistent pooled association with current DR severity. Associations with longer diabetes duration have also been reported, but the temporal sequence cannot be determined from the available data (12, 43). Within the proposed framework, this pattern is interpreted as a possible correlate of capillary rarefaction and cumulative ischemic burden. Avascular areas have also contributed to discrimination in composite scoring systems (42). These findings justify prospective evaluation of reduced capillary density and avascular areas as candidate markers of prevalent DR and current severity.

#### 5.2.2 Auxiliary markers: neoangiogenesis, megacapillaries, crossed-over vessels

Nailfold neoangiogenesis encompasses three main subtypes: bushy capillaries, ramified (branched) capillaries, and bizarre capillaries (54). In some diabetes-focused studies, similar changes are described as “neoformation,” which is interpreted broadly as equivalent to neoangiogenesis in this review. In a prospective study of 216 diabetic patients and 101 controls, Uyar et al. reported that bushy capillaries (p < 0.001), bizarre capillaries (p < 0.001), and neoformation-related changes (p < 0.001) were significantly more common in diabetic patients and associated with DR (38). Meta-analytic data report pooled odds ratios of 2.82 (95% CI: 1.65–4.80) for bushy capillaries and 4.61 (95% CI: 3.15–6.76) for neoformation-related neoangiogenesis in DR versus non-DR patients (12). Ramified capillaries remained associated with prevalent DR in a multivariable model restricted to pathological NVC parameters (OR = 8.35, 95% CI: 1.97–35.39) (55), although clinical confounders were not included in the model (51). A recent study further linked advanced DR specifically to bushy and crossed-over capillaries (35).

Megacapillaries had the highest pooled odds ratio among the evaluated NFC parameters (OR = 8.37, 95% CI: 3.45–20.32) (12). A case report described changes in megacapillaries after restoration of normoglycemia; whether this observation is reproducible remains uncertain (56). Crossing capillaries showed approximately 40% sensitivity and 80% specificity for PDR in one cross-sectional study; this estimate requires external validation (35, 57). These features form the proposed auxiliary component of the exploratory framework, although their temporal and mechanistic significance remains uncertain.

#### 5.2.3 Supplementary features: tortuosity, dilation, microhemorrhages, structural abnormalities

Capillary tortuosity is frequently observed in diabetic patients and correlates with disease duration and glycemic control; however, meta-analytic evidence shows inconsistent associations with DR severity, and mild tortuosity may also occur in healthy controls, limiting its specificity (12, 58). Capillary dilation has also been reported more frequently in diabetes; one cross-sectional study found dilated or giant capillaries in 51% of patients with diabetes and 35% of controls (P = 0.022) (59). Quantitative capillary width measurements show moderate discriminative performance for DR (AUC = 0.754) (41), yet mild ectasia is common even in patients without retinopathy. Extreme dilation classified as megacapillaries is discussed separately as an auxiliary marker (Section 5.2.2). Although tortuosity shows less consistent discrimination across DR severity grades, it remains independently associated with established DR in multivariable models (adjusted OR = 2.16), as confirmed by pooled meta-analytic data (12, 38). Its observation in some patients without clinically overt retinal disease supports further investigation of its temporal relevance in prospective cohorts. Microhemorrhages indicate increased capillary wall fragility and are more prevalent in DR, although their independent contribution to severity stratification requires further validation (38). Structural abnormalities including receding and angulated capillaries have been proposed as characteristic features of diabetic microangiopathy, with higher prevalence in patients with DR (43, 46). These supplementary features are best interpreted within the overall morphological pattern rather than in isolation.

#### 5.2.4 Composite assessment: multi-parameter performance

Current findings provide a rationale for prospectively evaluating multi-parameter approaches to NFC assessment. In individual cohorts, selected quantitative NFC parameters yielded AUC values above 0.80 for cross-sectional discrimination of prevalent DR or PDR (41, 53). One composite scoring system yielded an AUC of 0.745 for cross-sectional discrimination of prevalent DR (44), and another semiquantitative system showed associations between combined NFC abnormalities and current DR grade (42). In one cohort, adding capillary length to clinical factors increased cross-sectional discrimination of prevalent DR (41). Most available studies were cross-sectional and methodologically heterogeneous, with modest sample sizes. The reported AUCs and composite scores therefore reflect discrimination of existing DR or its severity, not prediction of onset or progression. These findings warrant prospective evaluation of NFC as an investigational approach to diabetic microvascular assessment (41).

## 6 A conceptual framework: NFC phenotypes and cross-sectional DR-related patterns

Cross-sectional studies report overlapping groups of NFC abnormalities. Background features occur across DR groups, whereas auxiliary and density-loss/avascular features have been reported more often in some cohorts with greater current severity. This pattern is hypothesis-generating and does not establish within-person progression.

### 6.1 Cumulative layering model: NFC abnormalities across static observational DR severity patterns

Within this hypothesis-generating model derived from cross-sectional observations, NFC abnormalities are provisionally organized into overlapping background, auxiliary, and density-loss/avascular components. The association of individual parameters with DR presence must be distinguished from their capacity to discriminate DR severity grades. These cross-sectional patterns offer a rational basis to test prospectively whether baseline NFC profiles or serial changes relate to subsequent DR onset and progression.

#### 6.1.1 Background signal:

Capillary tortuosity, mild dilation, and microhemorrhages are commonly reported background NFC abnormalities in diabetic patients, corresponding to endothelial dysfunction and increased capillary fragility (14, 38, 60). Meta-analytic evidence indicates these features do not reliably differentiate across DR severity grades (12). They are provisionally classified as background features because they have been associated with prevalent DR in some studies but show limited and inconsistent discrimination across current DR severity grades. Within the proposed framework, they constitute the background component (12, 35, 38, 61).

#### 6.1.2 Auxiliary morphological component

In cross-sectional datasets, neoangiogenesis, megacapillaries, and crossing capillaries have been reported in association with prevalent DR or current DR severity, although the findings are not uniform across studies (35, 38, 57). These abnormalities are provisionally grouped as morphological features potentially related to microvascular remodeling. Hypoxia-induced angiogenic signaling and progressive capillary wall injury provide a biologically plausible explanation, but their direct mechanistic and temporal relationships with NFC abnormalities have not been established (62). A case report described changes in megacapillaries following restoration of normoglycemia; however, whether this observation represents reproducible reversibility remains uncertain (56). Crossing capillaries showed approximately 77% specificity for prevalent PDR in one cross-sectional study, but this estimate requires independent validation (35). Quantitative capillary width and turbidity also differed across current DR categories, although they did not exhibit a consistent monotonic gradient from NPDR to PDR (41). Within this heuristic model, these auxiliary features are considered in combination with background abnormalities rather than as a confirmed stage of microvascular progression.

#### 6.1.3 Density-loss/avascular component

In some cross-sectional studies, reduced capillary density and avascular areas were more frequently observed in patients with more severe DR (12, 46). Within the proposed framework, these findings are provisionally interpreted as possible correlates of capillary rarefaction and impaired tissue perfusion. Chronic hyperglycemic microvascular injury and altered hemorheology provide a biologically plausible context for this interpretation, but do not establish a direct mechanistic correspondence between nailfold and retinal abnormalities (63, 64). Among the evaluated NFC parameters, avascular areas showed the most consistent pooled association with current DR severity (12). However, the available cross-sectional evidence cannot establish the temporal sequence, irreversibility, or direct causal relationship of these abnormalities with retinal non-perfusion.

**Figure 1:**
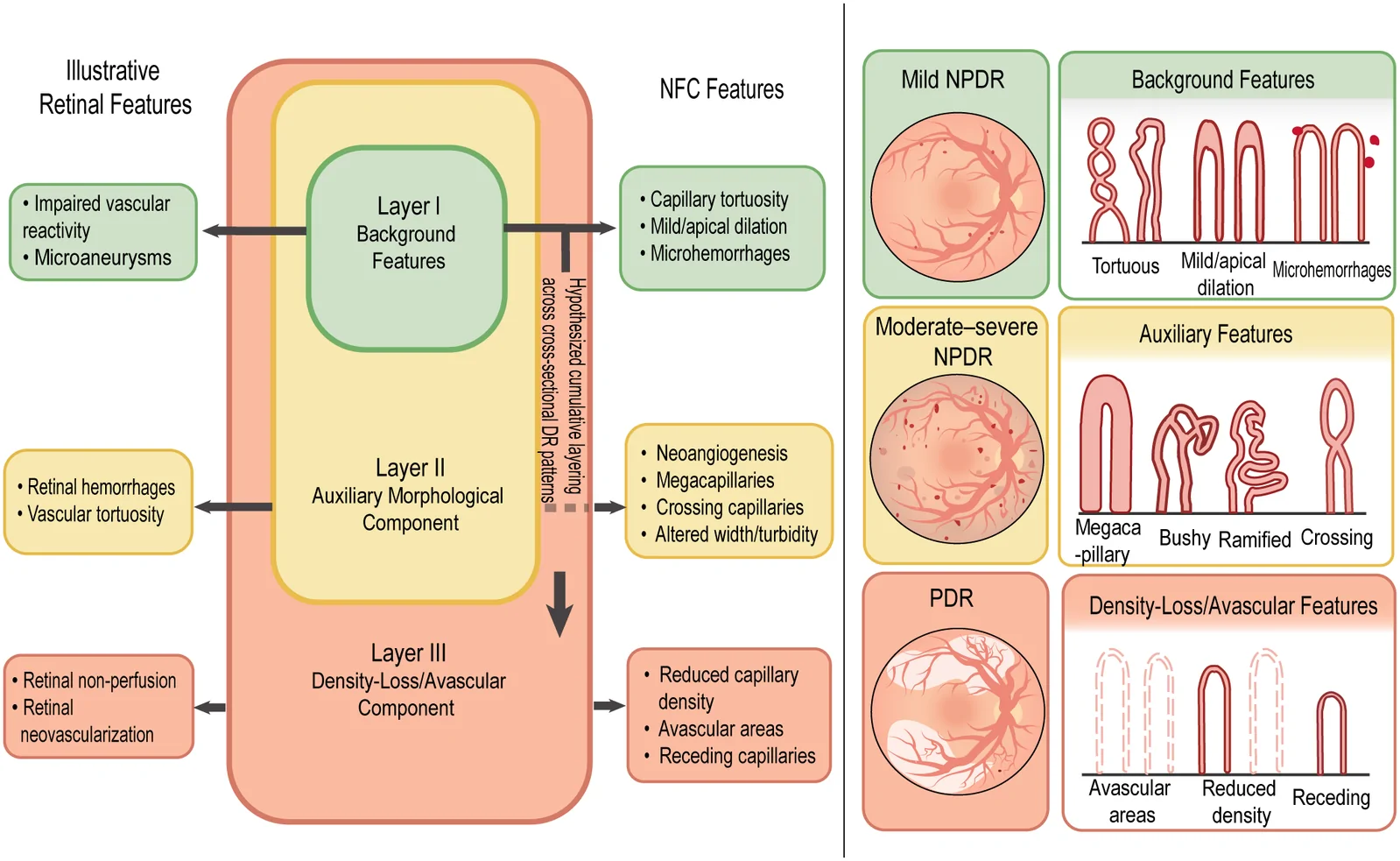
Hypothesis-generating cumulative layering model of nailfold capillaroscopic abnormalities and retinal microvascular features in diabetic retinopathy. Left panel: The central schematic provisionally organizes nailfold capillaroscopy (NFC) abnormalities reported across heterogeneous cross-sectional studies into three overlapping components. Layer I comprises background features, including capillary tortuosity, mild or apical dilation, and microhemorrhages. Layer II comprises auxiliary morphological features, including neoangiogenesis, megacapillaries, crossing capillaries, and altered capillary width and turbidity. Layer III comprises density-loss/avascular features, including reduced capillary density, avascular areas, and receding capillaries. The proposed NFC components are shown alongside illustrative retinal microvascular features reported in diabetic retinopathy (DR). Right panel: Representative NFC morphologies are displayed alongside schematic retinal findings labeled as mild non-proliferative DR (NPDR), moderate-to-severe NPDR, and proliferative DR (PDR). The alignment of the retinal illustrations with the proposed NFC components is provided solely for biological context and does not imply a validated one-to-one correspondence, within-person temporal progression, or direct mechanistic relationship between retinal and nailfold microvascular abnormalities. The cumulative layering model is a heuristic construct derived primarily from cross-sectional group-level observations and does not constitute a validated DR severity scale, clinical risk classification, or management framework. Prospective longitudinal studies are required to determine whether the proposed NFC patterns are reproducible and have temporal or incremental clinical relevance. All illustrations are schematic and not drawn to scale. DR, diabetic retinopathy; NFC, nailfold capillaroscopy; NPDR, non-proliferative diabetic retinopathy; PDR, proliferative diabetic retinopathy.

As a heuristic construct grounded in cross-sectional observations, the cumulative layering model provides a conceptual basis for prospective evaluation of the proposed pattern-based framework illustrated in **Figure 1**. Longitudinal studies are required to assess whether the proposed patterns are reproducible and have temporal relevance to incident or progressive DR.

### 6.2 Mechanistic basis: NFC as a potential peripheral correlate of retinal microangiopathy

Associations between NFC abnormalities and diabetic retinopathy are consistent with the systemic nature of diabetic microangiopathy (65). Endothelial dysfunction, basement membrane remodeling, and impaired capillary perfusion may affect both retinal and nailfold microvascular beds. In the nailfold, these processes may contribute to the capillary dilatation, tortuosity, microhemorrhages, reduced capillary density, and avascular areas reported in people with diabetes. However, shared systemic drivers do not imply identical mechanisms, morphological manifestations, or temporal progression in the two vascular beds.

#### 6.2.1 Neurovascular dysfunction

Neurovascular unit dysfunction is well established in the diabetic retina, where activation of microglia and Müller cells contributes to inflammation, barrier impairment, and altered perfusion (66–69). Equivalent neurovascular mechanisms have not been demonstrated directly in human NFC studies. Nevertheless, diabetic neuropathy and impaired neurogenic vasomotor control may contribute to altered digital microvascular tone and perfusion (70–72), potentially providing a plausible but indirect explanation for some NFC abnormalities. This hypothesis should be distinguished from the more established contribution of systemic endothelial injury.

#### 6.2.2 Local biomechanical context

The retina and nailfold differ substantially in their vascular architecture and local mechanical environments. Retinal vessels are embedded within neural tissue and exposed to intraocular pressure-related forces, whereas nailfold capillaries form superficial U-shaped loops that are influenced by digital tissue pressure and local hemodynamic forces (73, 74). These differences may modify the morphological expression of diabetic microvascular injury, including the patterns of capillary dilatation, tortuosity, and microhemorrhage observed by NFC. They may therefore partly explain why nailfold and retinal abnormalities are associated at the group level but do not necessarily show a one-to-one correspondence in individual patients.

NFC provides a view of peripheral microvascular injury that may be associated with retinal microangiopathy at the group level but does not reproduce retinal pathology. This interpretation remains a mechanistic hypothesis. Prospective studies are needed to determine whether NFC provides information beyond established retinal and metabolic assessments.

## 7 Exploratory translational framework for NFC phenotypes

### 7.1 Populations for prospective evaluation of NFC patterns

NFC has translational research potential in populations at elevated microvascular risk, including patients with type 2 diabetes without clinically confirmed DR, individuals with longer diabetes duration or persistently elevated HbA1c, those with established microvascular complications, and participants recruited from primary-care or resource-limited settings. These populations provide relevant cohorts for prospective evaluation of the temporal and incremental value of NFC alongside established retinal assessment.

### 7.2 Interpretive principles: from features to composite patterns

Within this hypothesis-generating framework, reduced capillary density and avascular areas are treated as candidate core parameters, auxiliary abnormalities represent more complex morphological patterns, and supplementary features constitute relatively common background findings. In individual cross-sectional cohorts, selected quantitative parameters have shown AUC values above 0.80 for discrimination of prevalent DR or PDR. A capillary density cutoff of <7.5 capillaries/mm yielded 95% sensitivity and 65% specificity for prevalent DR in one cohort, but this threshold has not been externally validated (53). Avascular areas have shown associations with current DR severity and diabetes duration, suggesting that they may reflect cumulative microvascular burden more closely than short-term glycemic status; this interpretation requires longitudinal confirmation (41, 75). NFC findings may be more informative when evaluated as composite morphological patterns rather than as isolated abnormalities. One multi-parameter score yielded an AUC of 0.745 for cross-sectional discrimination of prevalent DR (44). Selected quantitative NFC variables increased cross-sectional discrimination when added to clinical factors in another cohort (41, 75). Automated image analysis remains preliminary. One proof-of-concept study reported an AUROC of 0.84 for identifying diabetes from NFC images (39).

### 7.3 Exploratory pattern-based framework

The proposed three-tiered framework organizes NFC abnormalities reported across heterogeneous cross-sectional studies and provides structured hypotheses for prospective evaluation. It is intended as a conceptual synthesis rather than a validated temporal sequence, DR severity classification, risk-stratification system, or clinical management tool.

Pattern A: Background-predominant features. Background features, including mild tortuosity, limited capillary dilation, and microhemorrhages, are present without auxiliary or core abnormalities. These relatively common and potentially nonspecific findings may reflect systemic microvascular disturbance, but their temporal relationship with DR remains uncertain.

Pattern B: Mixed morphological features. Auxiliary abnormalities, including neoangiogenesis, megacapillaries, and crossing capillaries, coexist with background features. This combination may represent a more complex pattern of microvascular remodeling. Its relationship with DR should be evaluated after accounting for diabetes duration, cumulative glycemic burden, blood pressure, lipid status, renal function, and other established clinical factors.

Pattern C: Density-loss/avascular features. Core abnormalities, particularly markedly reduced capillary density and expanded avascular areas, coexist with background and auxiliary features, forming the proposed density-loss/avascular component of the framework. Whether this pattern corresponds to more advanced retinal microvascular involvement or has longitudinal relevance remains to be determined.

This framework is methodologically informed by quantitative capillaroscopy algorithms such as the CAPI-score in systemic sclerosis, which uses capillary density as a principal discriminative variable (76). However, the CAPI-score was developed for systemic sclerosis rather than diabetic microangiopathy and cannot be directly transferred to DR. Its density-first hierarchical structure is therefore considered only as a methodological reference for future consensus development, threshold derivation, and validation. Prospective studies are needed to determine whether the proposed NFC patterns are reproducible, demonstrate a temporal relationship with incident or progressive DR, and provide incremental information beyond established clinical factors. Until such evidence is available, the framework should be regarded solely as a hypothesis-generating model.

## 8 Discussion

### 8.1 Summary of key findings

Existing studies link selected NFC abnormalities with prevalent DR and current severity, although findings vary across parameters and methods. The cumulative layering model organizes these observations into background, auxiliary, and density-loss/avascular components for prospective testing. It is not a validated temporal sequence or clinical classification.

### 8.2 Strengths and translational research implications

The principal investigational relevance of NFC for DR research lies less in any individual morphological parameter than in its potential to reflect the cumulative microvascular burden of long-term hyperglycemic exposure — a phenotypic correlate of vascular memory that short-term metabolic indices such as HbA1c do not directly capture. These findings provide a rationale for evaluating NFC alongside established retinal and metabolic assessments, particularly when conventional metabolic indices may not fully reflect cumulative microvascular burden. The conceptual frameworks proposed in this review are intended to convert heterogeneous cross-sectional findings into a structured rationale for prospective testing and hypothesis-driven evaluation.

### 8.3 Methodological limitations: reproducibility and device heterogeneity

Inter-observer variability may represent a notable constraint on NFC reproducibility. Even among experienced examiners, consensus among ≥ 4/5 raters is achieved in only approximately 52% of cases; following standardized training, consensus rates remain below 70% (76). Assessment methods vary in reliability: qualitative pattern recognition is efficient but prone to subjectivity; semiquantitative scoring improves reproducibility when supported by standardized definitions (77); fully quantitative software-assisted methods may reduce subjectivity but require dedicated tools, as exemplified by the CAPI-score. Most DR–NFC studies use qualitative or semiquantitative methods with single-rater designs, which may increase measurement bias.

Device heterogeneity may further complicate cross-study interpretation. Studies vary widely in imaging device type, magnification (100× to 400×), finger selection, parameter definitions, and scoring systems. Performance differs considerably across device categories: NVC remains the reference standard for detailed morphological analysis, whereas dermatoscopy offers a more accessible imaging approach with lower grading reliability, and USB microscopy requires further validation for comprehensive quantitative phenotyping (41, 78, 79). In addition, skin color bias — reduced image contrast in individuals with darker skin tones — remains understudied and may partially limit cross-population generalizability. These limitations should be addressed in future multicenter validation studies.

### 8.4 Study design limitations: cross-sectional evidence and confounding

Most existing DR–NFC studies are cross-sectional, single-center, with modest sample sizes (59). Longitudinal evidence is accumulating but remains limited (80). Study endpoints also vary, with some studies examining DR presence and others evaluating associations with current disease severity, which complicates direct comparison of discriminative performance across individual NFC parameters (81). Adjustment for key confounders is often incomplete, which may affect internal validity and external generalizability. These limitations support treating the cumulative layering model as a hypothesis-generating synthesis of cross-sectional evidence. Its relationship with future DR onset or progression requires prospective evaluation.

### 8.5 Future directions

#### 8.5.1 Standardized evaluation protocols

DR-specific NFC evaluation frameworks should be developed using methodological precedents established in rheumatology (30, 34, 82). The development of the CAPI-score, involving multi-expert consensus, quantitative threshold derivation, hierarchical algorithm construction, and external validation, provides a methodological reference for DR-focused standardization (76). Future protocols should define image-acquisition procedures, device specifications, anatomical sampling sites, parameter definitions, reader training, and inter- and intra-observer reproducibility.

#### 8.5.2 Prospective longitudinal studies

Prospective cohort studies are required to determine whether baseline NFC abnormalities or serial changes are associated with subsequent DR onset or progression and to evaluate the temporal relevance of the proposed pattern-based framework (83). Such studies should establish whether the cumulative layering model reflects longitudinal microvascular evolution or merely the cross-sectional coexistence of heterogeneous abnormalities. Analyses should account for established determinants of DR, including diabetes duration, glycemic burden, blood pressure, lipid status, renal function, diabetes type, and treatment. Potential clinical applications should be considered only after reproducible longitudinal associations and external validity have been demonstrated.

#### 8.5.3 AI-automated interpretation

Future studies should evaluate DR-related outcomes as prespecified endpoints and systematically validate automated NFC interpretation in multicenter, real-world, and multi-device settings. Particular attention should be given to image quality, acquisition variability, model calibration, interpretability, and generalizability across patient populations and technical platforms (39, 84–88).

#### 8.5.4 Multidimensional integrated predictive models

If longitudinal associations are established, NFC parameters can be evaluated for incremental predictive value beyond established clinical models. Relevant assessments may include discrimination, calibration, net reclassification improvement, integrated discrimination improvement, and clinical utility. These analyses would help determine whether associations identified in cross-sectional datasets translate into meaningful longitudinal information and contribute to externally validated DR prediction models (89–91).

## 9 Conclusion

At present, NFC may be considered an investigational research approach for characterizing peripheral microvascular patterns associated with prevalent DR, rather than a clinically validated adjunct to conventional retinal assessment. Further evaluation requires standardized image acquisition and analysis, assessment of measurement reproducibility, prospective multicenter studies, and external validation of any incremental value beyond established clinical risk factors. NFC should not currently be used to define DR risk categories, determine screening intervals, prioritize retinal imaging, or guide ophthalmology referral, and established guideline-based retinal assessment should remain the standard of care. The cumulative layering model and pattern-based framework organize the available cross-sectional evidence into testable hypotheses but do not constitute validated temporal, severity, or clinical management frameworks. Prospective longitudinal studies are required to determine whether baseline or serial NFC abnormalities are associated with incident or progressive DR and provide reproducible information beyond established clinical factors.
