# Supplementary material for: Nailfold capillaroscopy in diabetic retinopathy: microvascular insights, clinical value, and translational challenges — a narrative review
Source: Front Endocrinol (Lausanne). 2026 Aug 13;17:1866310. doi: 10.3389/fendo.2026.1866310 (PMC13520592; doi:10.3389/fendo.2026.1866310)
Supplement: Supplementary file 1 [file Table1.docx]

**Supplementary Table 1. Overview of included studies evaluating associations between nailfold capillaroscopy parameters and diabetic retinopathy.**

| **Study (Year)** | **Population and DR Severity Assessed** | **Key NFC Findings and Discriminative Metrics** | **Statistical and Methodological Considerations** |
| --- | --- | --- | --- |
| Chang, 1997 (51) | Cross-sectional comparative study of 35 patients with diabetes mellitus, including 10 without retinopathy, 10 with background DR, and 15 with proliferative DR, together with 20 age-comparable controls without diabetes or vascular disease. | Capillary density showed no significant intergroup differences. Capillary tortuosity rose sequentially from controls, no DR, background DR to PDR (20 ± 12%, 23 ± 15%, 42 ± 19%, 68 ± 22%); background DR and PDR differed from controls (P<0.05), while PDR differed from background DR (P<0.01). Arterial limb diameter increased from 13 ± 1 μm (controls) to 17 ± 3 μm (background DR) and 18 ± 3 μm (PDR); venous limb diameter increased from 15 ± 3 μm to 20 ± 4 μm and 21 ± 3 μm (P<0.05 vs controls). Functional impairments preceded morphological alterations: resting capillary blood cell velocity declined across groups (0.36 ± 0.10 / 0.20 ± 0.11 / 0.19 ± 0.11 / 0.14 ± 0.05 mm/s), alongside reduced post-occlusive peak velocity (0.68 ± 0.23 / 0.42 ± 0.18 / 0.44 ± 0.24 / 0.32 ± 0.12 mm/s). Time to peak velocity lengthened progressively with DR severity (8.5 ± 2.5, 24.5 ± 14.9, 33.3 ± 15.2, 51.0 ± 16.1 s). | Dynamic nailfold capillaroscopy was performed on the left fourth finger following standardized acclimatization, including 5-min resting flow recording and a 1-min 200-mmHg arterial occlusion test to measure resting velocity, peak reactive hyperaemic velocity and time to peak velocity. Sodium fluorescein capillaroscopy was undertaken only in a small subgroup. Statistical analyses relied solely on unadjusted Wilcoxon rank tests. Key limitations: small cross-sectional cohort; unspecified diabetes subtype and glycaemic status; single-finger assessment; missing blinding and intra-/inter-rater reliability data; absence of multivariable adjustment, ROC analysis and validation. Gradually increasing diabetes duration across DR strata acts as a prominent confounder, hindering definitive attribution of NFC abnormalities to retinopathy severity alone. |
| Barchetta, 2011 (36) | Cross-sectional study of 49 patients with diabetes (21 T1DM and 28 T2DM) and 39 controls. Seventeen patients had DR: 13 had background/non-proliferative DR (6 T1DM and 7 T2DM) and four had proliferative DR (3 T1DM and 1 T2DM); 32 patients had no DR. | Compared with controls, diabetic patients had more frequent capillary abnormalities: reduced length (53.0% vs 28.2%, P=0.004), irregular distribution (14.2% vs 0%, P=0.02), abnormal morphology (79.6% vs 30.7%, P=0.0001), reduced density (14.2% vs 0%, P=0.02), ectasic loops (26.5% vs 12.8%, P=0.009), abnormal flow (32.6% vs 7.7%, P=0.004), oedema/exudates (12.2% vs 0%, P=0.03). NVC score ≥1 was seen in 59.1% diabetic patients vs 0% controls (P=0.0001), independently associated with diabetes (age/sex-adjusted OR 6.19, 95% CI 2.5–15.5). NVC score correlated with DR (Spearman r=0.42, P=0.0001; standardized β=0.30, P<0.02 multivariable-adjusted), identifying abnormalities in ~50% DR-free patients. T1DM linked to higher NVC scores and severer morphological defects versus T2DM. | NVC was assessed by one operator blinded to the participants’ clinical diagnosis using a predefined semiquantitative 0–3 scale for capillary length, distribution, morphology, density, diameter, haemorrhages, flow, oedema and exudates. Analyses included non-parametric comparisons, Spearman correlations, Mantel–Haenszel tests, and multivariable regression adjusted for age, sex and diabetes type, with post-hoc sample size estimation. Limitations involve a small single-centre cross-sectional sample, clinically indicated fluorescein angiography, absent intra/inter-rater reliability data, no validated diabetes-specific NVC cut-offs, and missing ROC analysis plus internal/external validation, constraining causal deduction and diagnostic interpretation. |
| Uyar, 2016 (38) | Prospective cross-sectional cohort: 216 T2DM patients (123 non-DR, 31 NPDR, 62 PDR) plus 101 healthy controls. DR diagnosed via full ophthalmic workup, fundus photography and OCT, graded by ETDRS standards. | Tortuosity rates were comparable in NPDR and PDR (80.6% each) and higher than non-DR (57.7%, P=0.002). Bushy capillaries and neoformation rose progressively from non-DR to NPDR to PDR (P<0.001, P=0.001), while capillary ectasia showed irregular distribution across groups (P=0.029); bizarre capillaries had no intergroup difference (P=0.372). In the reported logistic regression model, tortuosity remained associated with prevalent DR (OR = 2.11, 95% CI 1.05–4.22; P = 0.036). Cross-sectional discriminative performance was weak: AUCs were 0.615 (95% CI 0.540–0.689) for tortuosity, 0.606 (95% CI 0.528–0.683) for bushy capillaries, 0.595 (95% CI 0.517–0.673) for neoformation, 0.539 (95% CI 0.460–0.617) for microhemorrhages, 0.543 (95% CI 0.465–0.621) for bizarre capillaries, 0.531 (95% CI 0.452–0.609) for capillary ectasia, and 0.525 (95% CI 0.447–0.604) for aneurysms. Multiple NFC abnormalities were also associated with longer diabetes duration. | Large T2DM and control sample, blinded rheumatologist-performed ×200 videocapillaroscopy on eight non-thumb fingers (32 images per subject), standardized ETDRS retinopathy grading. Limitations: cross-sectional design, purely qualitative NFC without quantitative diameter/density metrics, no inter-rater reliability data. Logistic models unadjusted for longer disease duration and elevated HbA1c in DR patients; overrepresentation of PDR, uncorrected multiple comparisons, and internal-only ROC analysis weaken diagnostic and causal conclusions. |
| Kuryliszyn-Moskal, 2017 (93) | Cross-sectional study of 106 patients with T1DM and 40 healthy controls; 54 patients had microangiopathy and 52 did not. Retinopathy was identified by fundus photography in 45 patients. The DR-specific analysis was restricted to the microangiopathy subgroup (45 with vs nine without retinopathy); DR severity was not graded. | NVC abnormalities were detected in 81% of patients with T1DM. Severe abnormalities (score 3) were more frequent with than without microangiopathy (59.2% vs 13.4%). Within the microangiopathy subgroup, the mean NVC score was higher in patients with retinopathy (2.24 ± 1.00 vs 1.35 ± 1.09; P<0.001). No ROC-derived or other discriminative metrics were reported. | Standardized ×200 NVC of eight non-thumb fingers was evaluated by a single experienced investigator blinded to clinical history using a semiquantitative 0–3 score. Mann–Whitney U, χ², and Fisher’s exact tests were used. Limitations include the cross-sectional single-center design, absence of DR grading, multivariable adjustment and reliability analysis, and the small non-DR comparator group (n=9); coexisting nephropathy or neuropathy may have confounded the DR-specific association. |
| Bakirci, 2019  (47) | Single-center cross-sectional cohort of 64 T2DM patients (44 DR, 20 non-DR). DR diagnosed via ophthalmic assessment per AAO standards; no NPDR/PDR severity subgroup analyses or non-diabetic controls were conducted. | No qualitative or quantitative nailfold videocapillaroscopy (NVC) metrics differed significantly between DR and non-DR patients. DR patients showed numerically higher rates of ectatic capillaries (54.5% vs 40.0, P=0.281), giant capillaries (40.9% vs 25.0, P=0.219) and neoangiogenesis (6.8% vs 0, P=0.546); tortuosity prevalence was comparable (79.5% vs 75.0, P=0.749). Arterial, venous and apical capillary diameters also yielded non-significant intergroup differences (all P>0.1). | Blinded rheumatologist performed standardized NVC on all eight non-thumb fingers under controlled temperature/acclimatization; capillary diameters quantified from non-dominant 4th finger, with anonymized image scoring. Limitations: single-center cross-sectional design, small unbalanced DR/non-DR subgroups, no healthy controls or DR severity stratification, dichotomized morphological grading, missing inter-rater reliability data. All analyses were unadjusted without multiplicity correction; older age and longer diabetes duration in DR patients created confounding bias. |
| Jakhar, 2020  (92) | Single-center cross-sectional cohort of 96 T2DM patients and 40 healthy controls. The T2DM cohort was split into 46 subjects with microvascular complications and 50 without; secondary analysis contrasted 15 DR cases against 31 patients with non-retinal diabetic microangiopathies. | Any capillary abnormality was more prevalent in T2DM patients relative to healthy controls (80.2% vs 12.5%; P = 0.01). Compared to T2DM patients without microvascular complications, those with microvascular disease exhibited higher rates of meandering capillaries (78.3% vs 50.0%; P = 0.004), bizarre capillaries (17.4% vs 0%; P = 0.002), capillary dropouts (30.4% vs 10.0%; P = 0.012), and tortuosity (89.1% vs 72.0%; P = 0.035). No NFC metric, including mean capillary density (6.47 ± 0.91 vs 6.63 ± 1.08 capillaries/mm; P = 0.704), differed significantly between DR patients and those with other microvascular complications. No NFC abnormalities correlated with HbA1c or diabetes duration. | Age- and sex-matched healthy controls; standardized USB dermatoscopic examination of all 10 nailfolds, with quantitative capillary density measured in the fourth and fifth fingers bilaterally; images were captured by one observer and independently interpreted by two observers. Limitations include the single-center cross-sectional design, small and inconsistently reported DR subgroup, absent DR severity stratification, unadjusted bivariate analyses without multiplicity correction, and no reported reader blinding or inter-rater reliability. The DR-specific analysis compared patients with retinopathy against those with other diabetic microangiopathies rather than uncomplicated T2DM, limiting DR-specific inference. |
| Shikama, 2021  (57) | 108 Japanese outpatients with T2DM aged 40–75 years; 31 (28.7%) had DR. DR was classified via medical records using the Davis scheme into no DR, simple DR, pre-proliferative DR, or PDR; pre-proliferative DR and PDR were pooled for severity subgroup analysis. | The proportion of patients with DR rose across crossing-capillary tertiles (22.2%, 27.8%, 36.1%). Relative to the lowest tertile, multivariable-adjusted ORs for DR were 2.05 (95% CI 0.53–7.94) for the middle tertile and 4.33 (95% CI 1.16–16.21) for the highest tertile (P-trend = 0.028). The proportion of patients with combined pre-proliferative and PDR was 8.6%, 11.1%, and 19.4% across tertiles, with no significant overall association between crossing capillaries and DR severity (P = 0.585). | Standardized quantitative assessment with a DR-masked operator, two masked raters, and excellent inter-rater reliability (κ = 0.84); multivariable adjustment for major clinical confounders and supportive sensitivity analysis. Limited by the single-center cross-sectional design, medical-record-based DR ascertainment, only 31 DR cases, combined advanced-severity categories, wide CIs, potential model overfitting, and lack of validation in other populations. |
| Bohania, 2022 (37) | Observational cross-sectional cohort of 50 patients with diabetes (49 with T2DM and one with T1DM), including 17 patients with DR and 33 without DR. retinopathy severity was not graded or stratified. | Tortuous (56%), giant (46%), and cross-linked capillaries (44%) were the most frequent abnormalities. Compared with patients without DR, those with DR more frequently exhibited tortuous capillaries (94.1% vs 36.4%, P<0.0001), giant capillaries (88.2% vs 24.2%, P<0.0001), and avascular areas (35.3% vs 9.1%, P=0.047). Mean capillary length (67.06 ± 26.28 vs 98.18 ± 41.78 μm, P=0.007) and mean density (8.35 ± 2.23 vs 11.58 ± 2.50 capillaries/mm, P=0.0001) were lower in the DR group. | Small, single-center cross-sectional sample. NFC was performed at ×200 magnification on four fingers, with eight images obtained per participant; morphology was evaluated using modified Maricq criteria. Only unadjusted between-group analyses were conducted, while the DR group exhibited longer diabetes duration and higher HbA1c, which may cause confounding. Other limitations: absence of healthy controls, DR severity stratification, prospective follow-up, blinding, and inter-rater reliability assessment. Thus, diagnostic and causal inferences are restricted. |
| Abd EL-Khalik, 2022  (55) | Single-center cross-sectional study of 62 adults with T2DM: 36 without DR and 26 with DR, including 22 with NPDR and 4 with PDR. DR was determined by comprehensive ophthalmological assessment and classified according to ETDRS criteria. | Compared with non-DR patients, those with DR more frequently exhibited branched capillaries (69.2% vs 36.1%; P = 0.010), tortuous capillaries (69.2% vs 36.1%), and precapillary edema (46.2% vs 19.4%; P = 0.024), alongside larger mean capillary width (27.68 ± 5.70 vs 23.02 ± 3.29 μm; P = 0.001). Microhemorrhages and capillary length showed no significant intergroup differences. Multivariate logistic regression limited to pathological NVC markers identified branched capillaries (OR = 8.35, 95% CI 1.97–35.39; P = 0.004) and increased capillary width (OR = 1.35 per μm, 95% CI 1.14–1.61; P = 0.001) as independent correlates of DR. ROC analysis for capillary width yielded an AUC of 0.754 (95% CI 0.634–0.875), with a 27.8 μm cutoff delivering 42.3% sensitivity and 94.4% specificity for cross-sectional discrimination of prevalent DR. Global three-group comparisons across non-DR, NPDR and PDR revealed significant disparities for branched capillaries (P=0.026), tortuous capillaries (P=0.026), capillary width strata (P<0.001), and dilated apical capillaries (P=0.004). No linear increasing gradient of branched/tortuous capillaries was observed across DR tiers, while megacapillaries and dilated apical capillaries peaked in PDR patients. | Randomly sampled patients, standardized NVC assessment of 8–10 fingers using updated Maricq criteria, and ETDRS-based ophthalmological classification; limited by the single-center cross-sectional design, small sample—particularly only four PDR cases—and no reported blinding or observer-reliability assessment. DR patients had longer diabetes duration and higher HbA1c than non-DR patients, but the regression model included NVC variables only and did not adjust for these or other clinical confounders. Multiple comparisons were performed without reported correction, and the ROC cutoff was derived and evaluated in the same small cohort without internal or external validation, limiting severity-specific and diagnostic inference. |
| Raina, 2023  (44) | Pilot single-center cross-sectional study of 100 adults with T2DM: 54 with DR and 46 without DR. DR severity distribution was not reported; subgroup analyses were performed according to HbA1c levels. | Compared with non-DR patients, those with DR had lower capillary density (5.83 ± 0.72 vs 6.30 ± 0.89 loops/mm; P = 0.005) and higher numbers of microhemorrhages, giant capillaries, avascular areas, enlarged capillaries, tortuous capillaries, and abrogated/bushy capillaries (all P ≤ 0.004). An abnormal NFC score was more frequent in DR (51.85% vs 4.35%; P < 0.0001). A total NFC score >0 discriminated prevalent DR with an AUC of 0.745 (95% CI 0.648–0.827), 51.85% sensitivity, and 95.65% specificity. | Standardized 8-finger NFC using a smartphone-connected handheld dermatoscope (×10/×50) and semiquantitative scoring. Limitations include the pilot single-center cross-sectional design, modest sample size, absent DR severity stratification, unadjusted analyses without clinical confounder control, multiple uncorrected comparisons, imbalanced HbA1c subgroups, and no reported reader reliability or external validation. The AUC reflects cross-sectional discrimination rather than longitudinal prediction. |
| Lisco, 2023 (40) | 102 consecutive outpatients with T2DM; 31 (30.5%) had DR. DR was identified from clinical and fundoscopic information, but severity grading and NPDR/PDR subgroup data were not reported; no healthy controls were included. | Overall capillary density averaged 7.7 ± 1.6 capillaries/mm²; prevalent morphological abnormalities included tortuosity (65.7%), avascular areas (62.7%), bizarre capillaries (62.7%), microaneurysms (53.9%), and ectasias (49.0%). No significant differences in any NFC metric were observed between DR and non-DR patients, with no stratified quantitative DR data available. Patients with HbA1c ≥7% exhibited thicker, longer capillaries and higher rates of ectasias and microaneurysms. | Standardized 300× computerized nailfold video-capillaroscopy (CNVC) evaluating predefined quantitative and qualitative metrics in sequential T2DM outpatients. Limitations: single-center cross-sectional design, small DR subgroup (n=31), absent DR severity stratification, no healthy comparator group, imaging restricted to the 4th/5th fingers of the non-dominant hand, single-operator assessment with no inter-rater reliability data, unadjusted group comparisons, and no correction for multiple statistical tests. |
| Goydin, 2023 (94) | Single-center diagnostic study of 90 participants, including 31 patients with T2DM and NPDR, 29 with T2DM and PDR, and 30 clinically healthy controls without ophthalmic, endocrine, or vascular disease. | Linear capillary density, areal density, capillary length, and arterial and venous flow velocity declined progressively from controls to NPDR and PDR. Most intergroup differences reached P≤0.001. For NPDR versus controls: arterial velocity <512 μm/s (sensitivity 95.0%, specificity 100%, AUC 0.994); venous velocity <585 μm/s (sensitivity 88.3%, specificity 100%, AUC 0.982); areal density <38.4% (sensitivity 76.7%, specificity 86.7%, AUC 0.846). For PDR versus NPDR: arterial velocity <451 (sensitivity 100%, specificity 80.0%, AUC 0.941); venous velocity <441 (sensitivity 96.7%, specificity 76.7%, AUC 0.909); areal density <30.4% (sensitivity 96.7%, specificity 86.7%, AUC 0.963). Combined logistic models yielded apparent classification accuracies of 92.2% (NPDR) and 94.4% (PDR). | Quantitative NFC was performed with a KK-01 computerized capillaroscope to assess capillary morphology and flow; OCTA parameters enabled complementary retinal microvascular analysis. ROC-derived thresholds informed logistic regression (Nagelkerke R² = 0.766 for NPDR, 0.802 for PDR), and internal stability was tested via 20% jack-knife deletion. Limitations include a small single-center sample, healthy non-diabetic controls rather than non-DR T2DM participants, missing blinding and reproducibility data, and simultaneous threshold/model development in the same cohort, leading to spectrum and optimism bias. No external validation, calibration assessment, or CIs for diagnostic metrics were reported. Discrepancies existed in reported venous flow cut-offs, flow velocity units and regression formulas, limiting reliable interpretation. |
| Okabe, 2024  (41) | Multicenter cross-sectional cohort: 83 Japanese T2DM patients (30 non-DR, 24 NPDR, 29 PDR) plus 63 age- and sex-matched non-diabetic controls. DR graded via ETDRS using slit-lamp and dilated fundus photography; only the worse-affected eye was analyzed if bilateral severity differed. | T2DM patients displayed reduced capillary count (4.24 ± 1.57 vs 5.52 ± 1.28), shorter length (774 ± 569 vs 1445 μm), narrower width (23.6 ± 8.4 vs 28.4 μm) and higher turbidity vs controls (all P < 0.001). Across non-DR/NPDR/PDR groups, capillary count declined progressively (P = 0.02); length, width and turbidity also differed significantly (all P < 0.001) without a linear NPDR-to-PDR trend. After adjustment for age, sex, systolic blood pressure, hypertension, dyslipidemia, eGFR, and HbA1c, shorter capillary length, narrower width, and higher turbidity remained associated with prevalent DR, while all four quantitative parameters were associated with PDR. For cross-sectional discrimination of prevalent DR, AUCs were 0.66 for capillary count, 0.83 for length, 0.79 for width, and 0.78 for turbidity. The corresponding AUCs for PDR were 0.70, 0.83, 0.80, and 0.76. Adding capillary length to systemic clinical factors increased the DR AUC from 0.81 to 0.89 (P = 0.03); the increase in PDR AUC from 0.87 to 0.93 was not statistically significant. | Age/sex-matched non-diabetic controls, standardized ETDRS retinal grading, automated Kekkan-Bijin SC-10 quantitative NFC on the left 4th finger, covariate-adjusted logistic regression and DeLong AUC comparison. Limitations: cross-sectional design, small all-Japanese sample, single finger measurement, missing diabetes duration/smoking data. Only quantitative capillary metrics were analyzed (no morphological lesions like avascular zones or microhemorrhages); proprietary turbidity readouts hinder cross-device reproducibility. ROC/logistic models lacked external validation; glaucoma prevalence and lipid-lowering medication may introduce residual confounding. |
| Ahmad, 2024  (43) | Single-center cross-sectional study recruiting 262 Indian T2DM patients (144 non-DR, 118 DR including 102 NPDR and 16 PDR) plus 150 healthy controls. DR was confirmed via ophthalmic screening; subgroup comparisons were performed for DR vs non-DR and NPDR vs PDR in a cohort dominated by Fitzpatrick skin types IV–V. | Tortuosity, elevated capillary density, neoangiogenesis, microhemorrhages, avascular areas, crossing/meandering and receding capillaries were all significantly more prevalent in T2DM versus controls, and DR versus uncomplicated diabetes (all P < 0.001). ROC AUCs for cross-sectional discrimination of prevalent DR were 0.766 for tortuosity, 0.780 for increased capillary density, 0.724 for neoangiogenesis, and 0.728 for crossing/meandering capillaries. Neoangiogenesis, avascular areas, and receding capillaries were significantly more frequent in PDR than in NPDR (all P < 0.001). For discrimination of PDR from NPDR, AUCs were 0.720 for tortuosity, 0.744 for neoangiogenesis, 0.714 for microhemorrhages, 0.800 for avascular areas, 0.735 for crossing/meandering capillaries, and 0.736 for receding capillaries. Avascular areas showed the highest AUC for PDR discrimination. Cutoffs, sensitivities, and specificities were not reported. Capillary abnormalities correlated with longer diabetes duration and elevated HbA1c. | Large diabetic and control cohort; full nailfold qualitative assessment via DermLite DL4 handheld dermatoscope, standardized morphological definitions and ophthalmic DR grading. Limitations include single-center cross-sectional design, purely qualitative NFC readouts, unadjusted chi-square tests lacking multiplicity correction, absent reader blinding and inter-rater reliability data. Variable time lag between retinal and capillaroscopy assessments; darker skin (Fitzpatrick IV–V) impaired capillary visualization. ROC metrics were internally derived without cutoff/sensitivity data or external validation, weakening diagnostic interpretability. |
| Mahajan, 2024  (46) | Cross-sectional study of 100 adults with T2DM and established DR and 100 age- and sex-matched healthy controls. DR was graded using the Diabetic Retinopathy Disease Severity Scale: 50 NPDR cases (5 mild, 32 moderate, and 13 severe) and 50 PDR cases. No T2DM group without DR was included. | Compared with healthy controls, patients with T2DM and DR had higher prevalences of tortuosity, avascular areas, reduced capillary density, dilated, bushy, meandering, and angulated capillaries, and microhemorrhages (all P = 0.001); receding capillaries were also more frequent (P = 0.012). Across DR severity categories, tortuosity, avascular areas, reduced capillary density, bushy, meandering, angulated, and receding capillaries, and microhemorrhages differed significantly (all P ≤ 0.002), with the highest prevalences observed in PDR. In contrast, dilated capillaries, capillary dropouts, and subpapillary plexus visibility showed no severity-dependent differences (P = 0.202, 0.244, and 0.230, respectively). Several NFC morphological abnormalities were also associated with longer diabetes duration and higher HbA1c. | Standardized 10-finger NFC at ×20–×220 magnification, formal DR grading, and age- and sex-matched healthy controls; limited by single-center cross-sectional convenience sampling, no T2DM non-DR comparator, small NPDR subgroups, unadjusted analyses without multiplicity correction, and no reported reader blinding or inter-rater reliability. Comparisons with healthy controls cannot separate DR-specific abnormalities from general diabetic microvascular changes, while duration and glycemic control may confound severity-related associations. |
| Srivastava, 2024  (45) | 100 patients with diabetes (25 T1DM, 75 T2DM) and 100 age-/sex-matched comparison participants (26 prediabetes, 74 healthy controls); DR in 39 patients (9 T1DM, 30 T2DM); NPDR/PDR classification. | T1DM vs T2DM vs controls: mean NFC score 3.12 vs 2.05 vs 0.59; prediabetes: focal microhemorrhages only significant finding (p<0.05); DR vs non-DR: mean NFC score 4.05 vs 1.21; dilated 79.5%, tortuosity 64.1%, irregular distribution 56.4%, neoangiogenesis 51.3%, reduced length 33.3%, microhemorrhages 25.6%, reduced density 17.9% (all p<0.05); NFC score correlated with duration (rho=0.62, p<0.001), +0.27 per year; temporal milestones: dilated 9.5y, Megacapillaries  9.9y, focal microhemorrhages 9.9y, diffuse 14y; HbA1c>5.6%: mean NFC score 1.98 vs 0.59 (p<0.001) | Standardized Hsu NFC scoring, full nailfold examination under controlled environment, age-sex matched subjects, and comprehensive screening for retinopathy, nephropathy and neuropathy. Limitations: single-center cross-section, mixed T1/T2DM cohort, 26% baseline controls reclassified as prediabetic, concurrent multiple microvascular complications, no DR subgroup analyses. All analyses largely unadjusted without multiplicity correction; no blinding, inter-rater reliability or NFC score validation data. DR correlations are prone to confounding by diabetes subtype, disease duration, glycemia and coexisting microangiopathies. |
| Yan, 2025  (12) | Meta-analysis encompassing 12 cross-sectional studies with a total of 1,349 diabetic participants (628 patients with DR, 721 without DR). Six studies stratified DR severity, comprising 255 non-proliferative DR (NPDR) and 185 proliferative DR (PDR) cases. | Compared with diabetic patients without DR, those with DR exhibited significantly higher odds of bushy capillaries, neoformation, megacapillaries, tortuosity, microhemorrhages, meandering capillaries and avascular areas (OR range = 2.82–8.37). Among all evaluated NFC markers, only avascular areas showed a significant positive correlation with increasing DR severity (P = 0.04); no separate pooled OR values for isolated NPDR or PDR subgroups were reported in the meta-analysis. Quantitative capillary morphological metrics (length, width, density, venular diameter) showed no meaningful intergroup differences between DR and non-DR patients. | Study quality was assessed via the NIH tool; random-effects meta-analysis was used for highly heterogeneous outcomes (I² = 64–86%), with regional and DR severity subgroup analyses performed. Limitations included exclusive cross-sectional design, inconsistent NFC definitions and imaging devices, mostly unadjusted effect estimates, and limited severity-stratified data. |
| Raina, 2025  (42) | Pilot cross-sectional cohort of 54 T2DM patients with DR, graded by indirect ophthalmoscopy and fundus photography using ETDRS criteria. The cohort comprised 36 NPDR cases of varying severity, 21 PDR cases, two with advanced diabetic eye disease, and six with DME; no non-DR diabetic controls were included. | Avascular areas (P = 0.005), abrogated/bushy capillaries (P = 0.0007), count of involved digits, and total semiquantitative NFC score differed significantly across retinal disease tiers, with peak median values observed in advanced diabetic eye disease and DME. Capillary density, microhemorrhages, giant capillaries and enlarged loops showed no grade-dependent disparities (P = 0.400, 0.573, 0.224, 0.566). Pairwise subgroup comparisons of categorical abnormality proportions yielded no significant inter-retinopathy-group differences. | Retinopathy stratified by formal ETDRS grading; NFC imaging conducted on all eight fingers via smartphone-linked ×10/×50 handheld dermatoscope. Limitations: pilot cross-sectional design, no non-retinopathy diabetic control arm, tiny individual retinal severity subgroups, unadjusted statistical analyses lacking multiplicity correction, and no reported reader blinding or inter-rater reliability metrics for capillary assessment. |
| Elghany, 2025  (53) | Cross-sectional study of 120 patients with T2DM: 60 without DR and 60 with DR, including 15 each with mild NPDR, moderate NPDR, severe NPDR, and PDR. | Compared with non-DR patients, those with DR had lower capillary density (7.2 ± 0.86 vs 9.4 ± 1.12 capillaries/mm; P < 0.001), greater capillary diameter (24.5 ± 13.58 vs 17.1 ± 4.70 μm; P < 0.001), and more frequent irregular distribution, neoangiogenesis, dilated and giant capillaries, and microhemorrhages (all P ≤ 0.031). Across mild, moderate, and severe NPDR and PDR, density progressively decreased (8.0, 7.4, 6.9, and 6.3 capillaries/mm; P < 0.001), whereas diameter increased (17.8, 18.6, 26.0, and 35.5 μm; P < 0.001). Capillary density discriminated prevalent DR with an AUC of 0.937; a cutoff <7.5 capillaries/mm provided 95.0% sensitivity, 65.0% specificity, and 80.0% accuracy. For cross-sectional discrimination of PDR, capillary density had an AUC of 0.900; a cutoff <6 capillaries/mm provided 91.1% sensitivity, 73.3% specificity, and 86.7% accuracy. Capillary diameter had an AUC of 0.840 for PDR; a cutoff >22.5 μm provided 80.0% sensitivity, 80.0% specificity, and 80.0% accuracy. | Age-sex balanced DR/non-DR groups with predefined sample size; standardized 8-finger NFC (×50/×200, four images per digit) and full ophthalmic DR grading. Limitations: single-center cross-section, artificial balanced DR subgroups inconsistent with real-world prevalence, missing reader blinding and reliability data. DR and non-DR patients differed markedly in duration, HbA1c and renal markers, yet multivariate models only incorporated capillary metrics without clinical confounder adjustment. Multiple uncorrected comparisons and internally validated ROC cutoffs restrict diagnostic inference. |
| Prakashey, 2025  (59) | Single-center cross-sectional study enrolling 100 T2DM patients and 100 age-sex matched healthy controls. T2DM cohort included 61 non-DR and 39 DR subjects (21 mild, 14 moderate, 3 severe NPDR, 1 PDR). DR was diagnosed via fundoscopy without explicit grading standards; no severity-stratified NFC analyses were conducted. | Only receding capillaries differed significantly between DR and non-DR T2DM patients (89.7% vs 21.3%, P = 0.001). Mean capillary density trended lower in DR (6.57 ± 0.71 vs 6.78 ± 0.79 capillaries/mm, P = 0.171), while all other qualitative NFC markers showed non-significant intergroup gaps (all P > 0.05). Relative to healthy controls, the overall T2DM group exhibited reduced capillary density (6.70 ± 0.76 vs 7.01 ± 0.77 capillaries/mm, P = 0.004) and elevated prevalence of multiple capillary morphological defects. | Standardized ×200 USB dermatoscopy with bilateral 4th/5th finger density counting, plus integrated qualitative and MDAD scoring, and matched healthy controls. Limitations: single-center cross-sectional design, small DR sample with minimal severe/PDR cases, unstated DR grading rules, no severity subgroup NFC comparisons. All tests were unadjusted without multiplicity correction or adjustment for duration/HbA1c; reader blinding and inter-rater reliability data were missing. Defining positivity by any single abnormal nailfold may overestimate incidental microvascular lesions, and MDAD scoring lacked diabetic cohort validation. |
| Zavareh, 2026 (35) | Single-center case–control study of 150 patients with T2DM, comprising 75 patients without DR and 75 with DR. The DR group included 56 patients with NPDR and 19 with PDR. | Compared with non-DR patients, those with DR more frequently exhibited ectatic (72.0% vs 50.7%; reported adjusted P=0.004), tortuous (66.7% vs 37.3%; P<0.001), and crossed-over capillaries (40.0% vs 22.7%; P=0.022). Crossed-over capillaries were more frequent in PDR than NPDR (63.2% vs 32.1%; reported P=0.017). Bushy capillaries were also reported to be more frequent in PDR (42.1% vs 28.6%; reported P=0.021), although this P-value appears inconsistent with the tabulated frequencies. For DR discrimination, tortuosity yielded 66.7% sensitivity, 62.7% specificity, and an AUC of 0.647, whereas crossed-over capillaries yielded 40.0% sensitivity, 77.3% specificity, and an AUC of 0.587. Ectasia was reported to have 28.0% sensitivity, 49.0% specificity, and an AUC of 0.613; however, these indices conflict with its reported prevalence and may reflect coding or reporting error. Overall, standalone discrimination was limited. | Standardized ×200 NFC of eight non-thumb fingers was evaluated by a blinded rheumatologist using Atlas-based criteria. The study included an a priori sample-size calculation, ROC analysis, and reported adjustment for age, sex, diabetes duration, BMI, and HbA1c, although adjusted effect estimates were not provided. Limitations included the single-center case–control design, artificially balanced DR/non-DR groups, unequal NPDR/PDR samples, no reader-reliability assessment, and no internal or external validation. Several results were internally inconsistent, including ectasia frequencies across Tables 2 and 3, its reported diagnostic indices, the bushy-capillary severity P-value, and the directions of associations with diabetes duration and BMI. These discrepancies limit interpretation of the severity and diagnostic findings. |

This table summarizes all included studies, including study populations, DR ascertainment and severity grading, principal NFC findings, cross-sectional discriminative performance, and major statistical and methodological considerations. Studies are arranged chronologically by year of publication; studies published in the same year are ordered according to their citation sequence in the main text. AUCs, 95% CIs, cutoffs, sensitivities, specificities, predictive values, and accuracies are presented only when directly reported in the original articles. Reported associations and ROC results reflect cross-sectional relationships with prevalent DR or discrimination of current DR status and severity and should not be interpreted as evidence of longitudinal prediction of incident or progressive DR. Cross-study comparisons should be interpreted cautiously because of heterogeneity in study populations, DR grading standards, imaging devices, NFC definitions, and analytical methods. The absence of discriminative performance measures in an individual study row indicates that these measures were not reported in the corresponding article.

Abbreviations: AUC, area under the receiver operating characteristic curve; CI, confidence interval; DME, diabetic macular edema; DR, diabetic retinopathy; ETDRS, Early Treatment Diabetic Retinopathy Study; NFC, nailfold capillaroscopy; NPDR, non-proliferative diabetic retinopathy; NVC, nailfold videocapillaroscopy; PDR, proliferative diabetic retinopathy; ROC, receiver operating characteristic; T1DM, type 1 diabetes mellitus; T2DM, type 2 diabetes mellitus.
